# Bedside Flexible Cystoscopy-Guided Ureteric Stent Insertion for Early Control of Sepsis

**DOI:** 10.7759/cureus.37841

**Published:** 2023-04-19

**Authors:** Sima Patel, Ridwaan Sohawon, Alexandros Georgiou, Suresh Ganta

**Affiliations:** 1 Surgery, New Cross Hospital, Wolverhampton, GBR; 2 Urology, Walsall Manor Hospital, Walsall, GBR

**Keywords:** flexible cystoscopy, ureteric stent, septic patient, infection control, sepsis

## Abstract

Sepsis is a life-threatening condition that needs the clinician to act quickly and swiftly in order to provide the best medical outcome for the patient. Sepsis can lead to multi-organ dysfunction, which is not only a risk to life but also utilizes multiple resources within the healthcare services. The management of any infection is reliant on two major factors: antimicrobial therapy and source control. We present two cases where source control, in the form of a ureteric stent insertion, was performed at bedside via the use of flexible cystoscopy to provide source control in the management of a septic patient.

## Introduction

Sepsis is defined as organ dysfunction and is a major risk to life. Considered a medical emergency, sepsis requires prompt treatment for optimal patient outcomes with antibiotic administration recommended internationally, within the initial hour of recognition and management, in any patient who presents with life-threatening septic shock [1].

Sepsis encompasses a range of symptoms that can lead to harm to the host. Symptoms associated with a septic patient include hypotension, tachycardia, hypo- or hyperthermia, or a change in the patient's respiratory rate. The official definition of sepsis as per the Third International Sepsis Taskforce is a “life-threatening organ dysfunction due to dysregulated host response to infection” [2].

Antibiotics are the cornerstone of sepsis management, but source control is another important feature in the management strategy when treating a septic patient. Marshall et al. describe source control as the physical control of the foci of infection and the restoration of optimal function and quality of life [3]. The theory of source control along with antibiotic use is that once the focus of infection is physically removed, the ongoing inflammatory process that leads to the symptoms of sepsis stops, allowing the patient to recover with antibiotics and appropriate resuscitation.

With urosepsis forming 25% of all hospital admissions and septic shock secondary to urosepsis carrying a 20% to 40% mortality rate during admission, prompt recognition and management is essential to good patient outcomes [4]. An infected obstructed kidney is one of the most common urological causes of sepsis, which requires immediate intervention to prevent renal injury and patient decline due to sepsis.

When managing patients for urosepsis, it is important to look for the sources of infection, both medical and surgical, such as urolithiasis, abscesses, or urinary tract infections. Common tests and investigations include urinary analysis, blood tests for biochemical markers in keeping with infection (such as a raised C-reactive protein or white blood cell count), and imaging in the form of ultrasonography or computed tomography (CT).

Intervention for an obstructed infected kidney is focused on decompression of the obstructed system, either via retrograde ureteric stent or insertion of a percutaneous nephrostomy, along with the use of antibiotics. The choice between a ureteric stent and a percutaneous nephrostomy is based on the availability of resources [5].

The insertion of a percutaneous nephrostomy is traditionally carried out through interventional radiology, while the placement of a retrograde ureteric stent is traditionally carried out via the use of fluoroscopy under general anesthetic on the emergency theater list by the urologist. The placement of a ureteric stent under local anesthetic has been described in the literature, although this has generally been carried out in an outpatient setting as opposed to ward-based care.

The advantages of insertion of a retrograde ureteric stent under local anesthesia include avoiding both potential admission and the patient having to undergo a general anesthetic; it can be done in a clinic setting as opposed to utilizing the emergency list; and it also has the potential to save both costs and time, which is vital for a service already stretched to breaking point [6].

The insertion of a percutaneous nephrostomy or a ureteric stent is sometimes unavoidable in obstructive uropathy [7]. We present two cases of individuals who presented to a district general hospital within the National Health Service (NHS) in the United Kingdom, were treated for urosepsis secondary to an obstructing calculus, and were managed via the use of both antibiotics and the placement of a ureteric stent, done under local anesthesia at the bedside, for source control of the septic patient.

## Case presentation

Case study one

We present a 46-year-old lady who presented with a 12-hour history of right-sided abdominal pain, described as loin to groin, with associated pyrexia. The patient was reported to be fit and well prior to this presentation and had no significant comorbidities. On examination, the abdomen was found to be soft and nontender, with no focal masses or areas of tenderness. Tachycardia and hypotension were noted, with a heart rate of 102 beats per minute and a systolic blood pressure of 98 millimeters of mercury (mmHg), when observations were taken. The patient’s urine analysis is shown in Table 1.

**Table 1:** Urine analysis on presentation taken in the accident and emergency department.

Parameter	Result
Blood	4+
Leucocytes	1+
Nitrates	2+
Ketones	1+

The patient’s blood results on initial presentation are shown in Table 2.

**Table 2:** Blood results on the initial presentation showing raised inflammatory markers in keeping with a potential infection.

	Result	Normal range
Sodium	139	133–146 mmol/L
Potassium	4.2	3.5–5.3 mmol/L
Urea	7.8	2.5–7.8 mmol/L
Creatinine	80	45–84 mmol/L
Estimated glomerular filtration rate	76	60–200 ml/min/bsa
C-reactive protein	5	0–5 mg/L
White cell count	24.7	115–160 g/L
Neutrophils	23.7	1.8–7.7 (10^9^/L)
Hemoglobin	142	115–160 (g/L)

As shown in Table 2, this patient had raised inflammatory markers with a mild decline in renal function combined with the aforementioned clinical findings on examination. A clinical diagnosis of sepsis was made for the patient. The source of infection was thought to be urinary, so appropriate investigations in the form of a non-contrast computed tomography (CT) were requested and management started.

**Figure 1:**
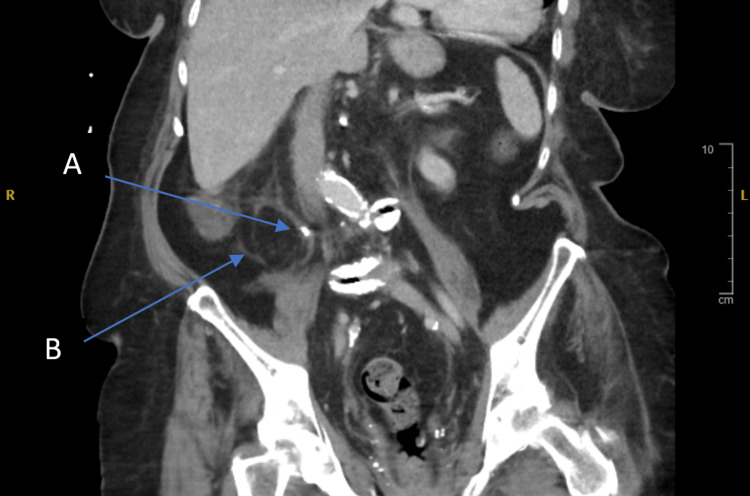
Coronal view showing a proximal ureteric stone (A) with associated fat stranding noted around the right ureter (B).

A non-contrast computed tomography of the kidneys, ureters, and bladder was performed and showed a large 11 mm proximal ureteric stone with associated hydronephrosis, as shown in Figure 1.

**Figure 2:**
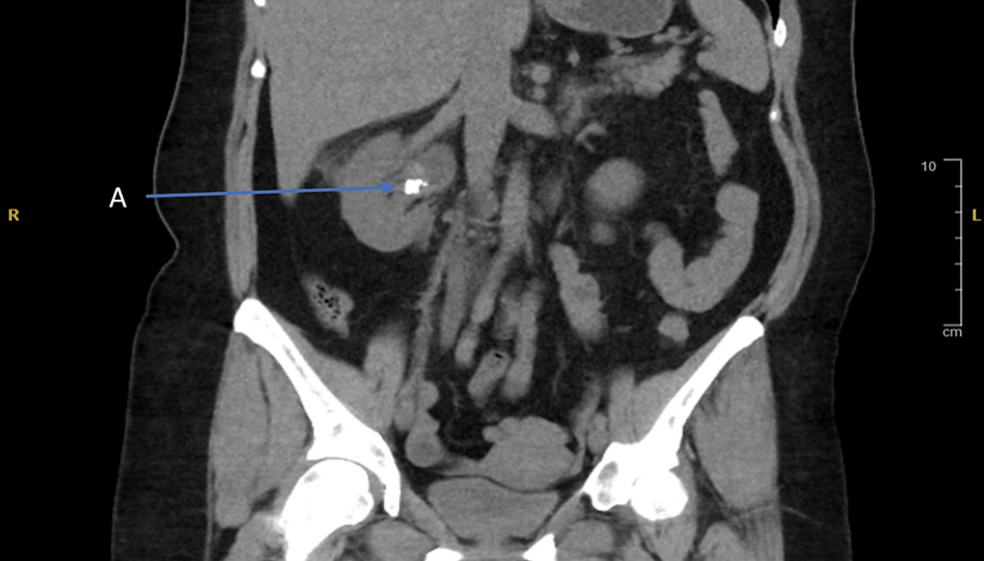
Extent of calculi size with the tip extending into the renal pelvis (A). Note the brightness of the calculi in contrast to the surrounding tissue, as this is a non-contrast scan.

As Figure 1 shows, there is extensive fat stranding around the right ureter, which can be due to many reasons such as inflammation, infection, or ischemia. Figure 2 shows a coronal section of the CT scan showing the ureteric stone extending into the renal pelvis.

**Figure 3:**
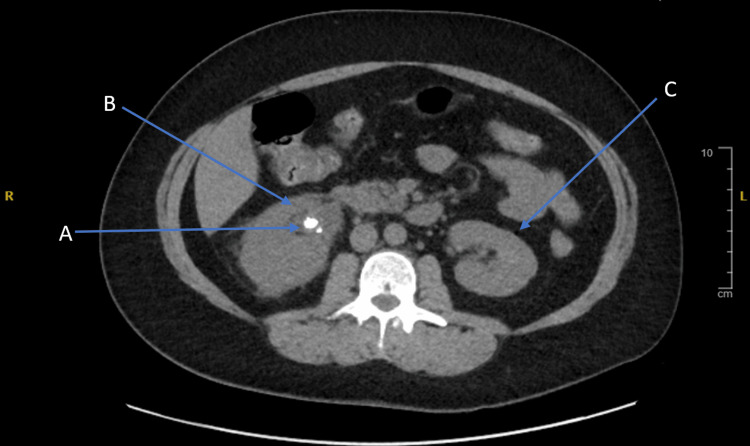
Right ureteric stone (A) with associated hydronephrosis (B) with a normal-looking left kidney (C).

The non-contrast computed tomography of the kidneys, ureters, and bladder was able to show the stone as well as the associated upstream hydronephrosis caused by the obstructing calculi, shown in Figure 3.

The patient was managed via parental antibiotics, and the insertion of a right retrograde ureteric stent was performed under local anesthetic (using 10 ml of lidocaine gel) on the ward, using a disposable flexible cystoscope. On insertion of the retrograde ureteric stent, purulent material was noted to be coming from the right ureter after the insertion of the ureteric stent, which allowed for decompression of the urinary tract.

An abdominal film was taken after the insertion of the ureteric stent to assess the placement of the stent. The patient remained stable after the procedure and was discharged two days following admission with a course of oral antibiotics and planned elective definitive management of the ureteric stone, aimed to occur within six to eight weeks. The ureteric stent was removed at the time of definitive stone management, eight weeks post-admission.

Case study two

The second case we present is that of an 85-year-old female patient who presented to the accident and emergency department with increasing confusion, pyrexia, and hypotension. Co-morbidities of the patient included type 2 diabetes, hypertension, and bilateral cataracts. Urine analysis of the patient was not performed due to the patient not passing urine while in the accident and emergency department. The patient did have a urethral catheter inserted as part of the sepsis management strategy in place with the trust hospital to monitor fluid balance and help guide further intravenous fluids. However, a “clean catch” sterile sample was not obtained on the insertion of the catheter. Blood results on initial presentation to the emergency department show features in keeping with an acute kidney injury and possible infection. Blood results on admission are shown in Table 3.

**Table 3:** Blood results on the initial presentation showing raised inflammatory markers and a decline in renal function suggestive of acute kidney injury, which is an example of organ dysfunction secondary to sepsis.

	Result	Normal range
Sodium	136	133–146 mmol/L
Potassium	3.5	3.5–5.3 mmol/L
Urea	9.8	2.5–7.8 mmol/L
Creatinine	129	45–84 mmol/L
Estimated glomerular filtration rate	33	60–200 ml/min/bsa
C-reactive protein	154	0–5 mg/L
White cell count	3.7	115–160 g/L
Neutrophils	3.1	1.8–7.7 (10^9^/L)
Hemoglobin	115	115–160 (g/L)

**Figure 4:**
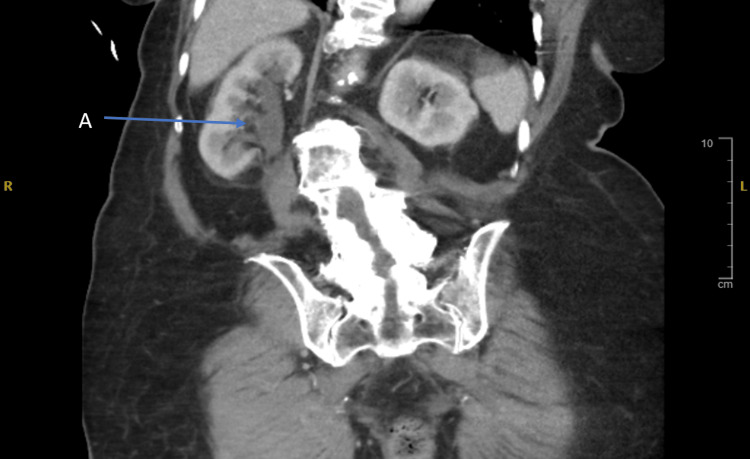
Coronal view of contrasted computed tomography of the abdomen and pelvis, which showed right-sided hydronephrosis (A).

A contrasted computed tomography of the thorax, abdomen, and pelvis was performed, which showed mild dilatation of the right renal pelvicalyceal system and proximal ureter secondary to a calculus measuring 6.5 mm seen lodged within the proximal third of the ureters. Prominent perinephric and periureteric fat stranding amounting to small fluid collections in the right retroperitoneal region was noted, which was suggestive of acute obstructive uropathy. Figure 4 highlights the right-sided hydronephrosis and the normal appearance of the left kidney.

**Figure 5:**
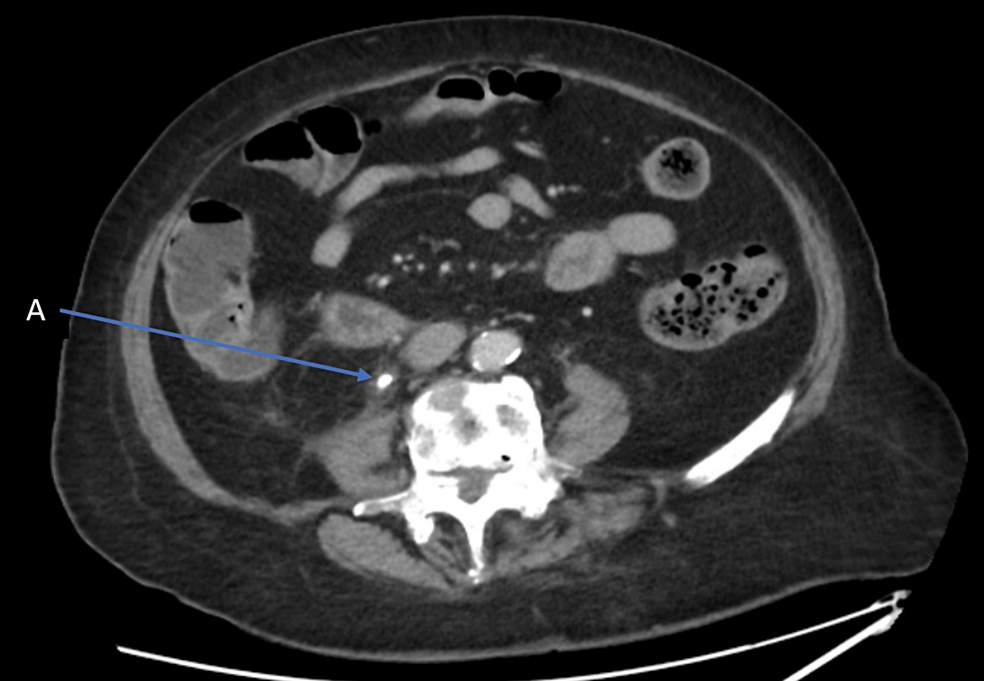
Axial view of contrasted computed tomography of the abdomen and pelvis showing impacted right-sided pelvic-ureteric junction stone (A).

As the patient was clinically unwell, a contrasted computed tomography of the thorax, abdomen, and pelvis was performed; however, an impacted stone was still noted distal to the right-sided hydronephrosis as shown in Figure 5.

**Figure 6:**
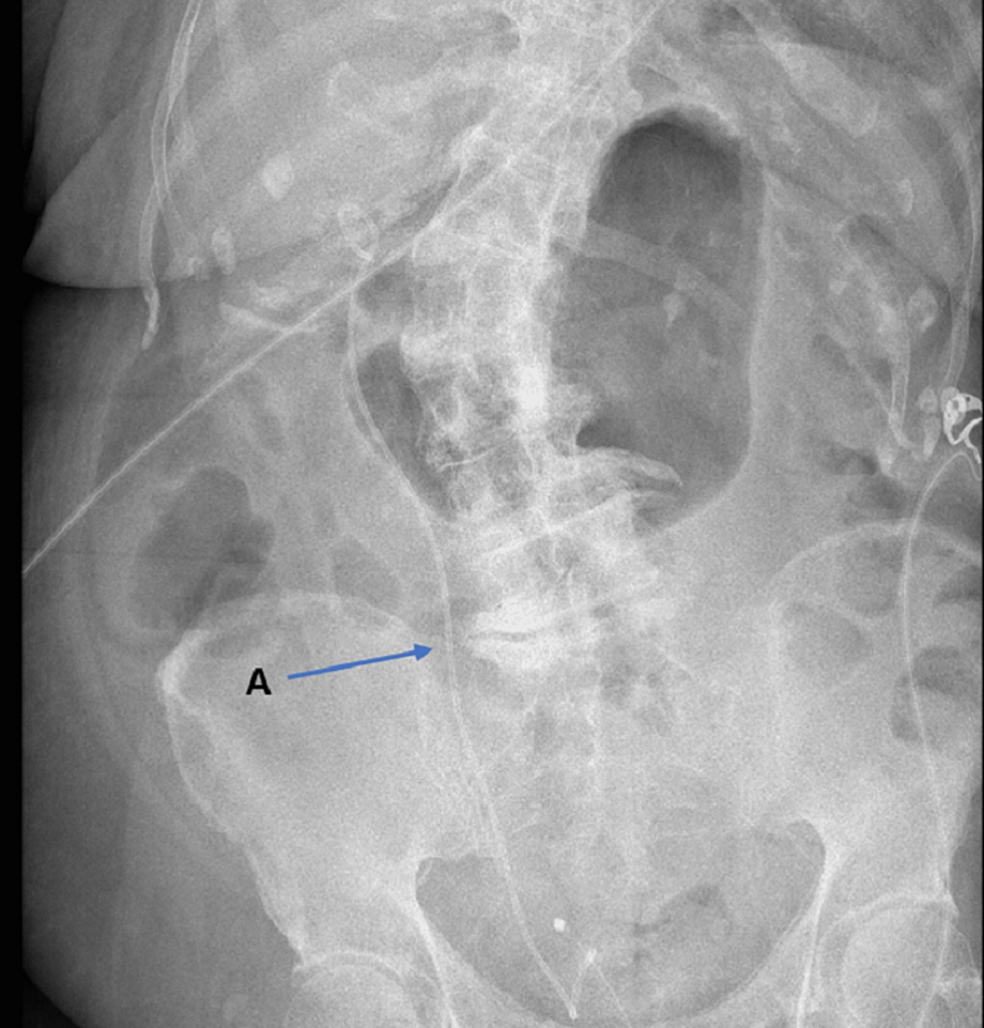
Satisfactory placement of a right-sided ureteric stent (A) on an abdominal x-ray.

The patient became hemodynamically unstable within the department and was subsequently transferred to the high-dependency resuscitation area within the accident and emergency department, where the urology team was contacted. The urology team inserted a right-sided retrograde ureteric stent at the bedside through the use of a disposable flexible cystoscope and local anesthetic (lidocaine gel). An abdominal x-ray was performed to assess the position of the abdominal stent, as shown in Figure 6.

The patient remained hypotensive in spite of intervention, parenteral antibiotics, and fluid resuscitation and was transferred to the intensive care unit for ongoing management and support with definitive management of the stone planned as an outpatient.

## Discussion

Equipment, setup, and technique

The equipment required for the insertion of a ureteric stent on the ward via the use of a disposable, flexible cystoscope is shown in Table 4.

**Table 4:** The equipment required for ward-based insertion of a ureteric stent for early sepsis control. *Ambu aView^TM^ 2 Advance brand used for the monitor. **Ambu aScope^TM^ 4 Cysto brand used for flexible cystoscope. ***Catheter packs contain sterile cotton balls, sterile gauze, sterile drapes, two sterile pots, and a sterile tray. ****10 ml Lidocaine gel used for local anesthetic.

Equipment required for insertion of the ureteric stent on the ward under local anesthetic
One drip stand
One monitor for flexible cystoscope*
One flexible cystoscope**
One intravenous fluid-giving set
One liter (l) of sodium chloride 0.9%
Catheter pack***
10 ml 0.9% Sodium chloride
Local anesthetic****
One soft tip hydrophilic guidewire
One ureteric stent
One ureteric stent pusher

The setup for the technique of inserting a ureteric stent on the ward via the use of a flexible cystoscope involves mounting the monitor onto the drip stand to allow both the patient and the operator to view the screen comfortably. Attach the 1 l bag of sodium chloride 0.9% to the drip stand and attach the giving set to the end of the bag as would be done in routine use. Prior to the preparation of the sterile field, ensure all equipment is working and fluid is flowing through the giving set at the desired rate and can be controlled by the operator. A "dirty" nonsterile assistant will be required to connect the 0.9% sodium chloride to the scope and help control the flow to ensure the operator maintains sterility throughout the procedure. Using an aseptic, non-touch technique, open the catheter pack and prepare a sterile field. Decant the 10 ml of 0.9% sodium chloride into one of the sterile pots, using the second pot to discard nonsterile cotton balls once used. Clean the external urethral meatus using sterile cotton balls and drape the area to create a sterile field for working as done when preparing to insert a urethral catheter. Insert 10 ml of local anesthetic into the external urethral opening and waiting the appropriate amount of time for the local anesthetic to take effect. Insert the flexible cystoscope under direct vision and fill the bladder using the 0.9% sodium chloride fluid. Perform cystoscopy and identify both ureteric orifices (UO). Once both UO have been identified or the bladder has sufficiently filled without causing overdistension, stop irrigation. One cystoscopy has been performed; insert the hydrophilic soft tip guidewire through the port-a three-way tap can be used here. Insert the hydrophilic, soft tip guidewire into the appropriate UO until resistance is felt in the kidney. Remove the cystoscope entirely, care must be taken to avoid dislodging the guidewire. Reinsert the cystoscope alongside the guidewire. Pass the ureteric stent over the guidewire as would be done routinely when inserting a ureteric stent, the ureteric pusher used after insertion of the ureteric stent to push the ureteric stent up the ureter sufficiently to ensure the correct position of the ureteric stent, suggested by coiling of the distal end of the ureteric stent near the bladder neck. Empty the bladder and request post-procedure imaging, such as an abdominal x-ray.

Patient discomfort can be a limiting factor in this technique; therefore, analgesia prior to the procedure may help overcome this. However, if the patient is unable to tolerate this method due to discomfort, the procedure should be abandoned and the patient considered for ureteric stent insertion under general anesthetic or percutaneous nephrostomy insertion.

Discussion

The aims of a management plan for septic patients are early recognition, immediate support, and antibiotic therapy to improve patient morbidity and mortality [8]. Delays to antimicrobial therapy increase the mortality of a patient [9]; however, source control must be considered to eradicate the infective foci and allow for antimicrobial therapy to take effective [10].

An obstructed infected kidney necessitates immediate action in the form of parental antibiotics and decompression of an infected obstructed system. Decompression can be in the form of percutaneous nephrostomy insertion via interventional radiology or the insertion of a retrograde ureteric stent, commonly performed by the urology team within a theater setting under general anesthesia. Rüddel et al. performed a secondary review of a clustered randomized control trial, which showed that both antimicrobial treatments, as well as source control, are time-critical when treating a septic patient [11].

Percutaneous nephrostomy insertion and retrograde ureteric stenting have shown to have comparable success rates in obstructive uropathy secondary to urolithiasis; however, radiation exposure, length of stay in the hospital, and associated costs were higher in those with percutaneous nephrostomies when compared to ureteric stenting [7].

Endoscopic procedures are not a new concept. The first flexible cystoscopy was described and performed in 1981 by Wibur using a flexible choledochoendoscope inserted into the bladder, making flexible cystoscopy a relatively new endoscopic procedure when compared to other endoscopic procedures [12,13]. Flexible cystoscopy can be used for both the investigation and management of a range of symptoms and conditions, so it has become a common investigation within urology. In the outpatient setting, common uses for flexible cystoscopy include insertion of ureteric catheter, initial investigation for hematuria, and investigating the causes of frequent urinary tract infections, to name a few, and as such, training around the use of flexible cystoscopy begins very early on in the urologist’s career.

The insertion of a ureteric stent is a common procedure within urology, as demonstrated by the fact that it forms one of the core procedures required to be performed at a high level for higher specialist surgical training in urology within the United Kingdom. With increasing patients presenting to the accident and emergency department and limited resources [14], the insertion of retrograde ureteric stents, using local anesthesia, performed on the ward, has the potential to reduce admission times and costs associated with obstructive uropathy requiring theaters for acute management of the septic patient while providing optimal patient care.

Sivalingam et al. demonstrated the placement of a ureteric stent successfully and safely within an office setting, while Kose et al. describe the replacement of a JJ ureteric stent (Boston Scientific Corporation Limited, Marlborough, MA, USA) in female patients without the use of fluoroscopy using only a cystoscope and a guidewire technique [15,16]. The technique described by Kose et al. allow the concept of ureteric placement on the ward using local anesthesia and flexible cystoscopy to be a possibility, as the technique is not dissimilar.

Sinha et al. describe the placement of ureteric stents using flexible cystoscopy within the outpatient setting with an 85.5% procedure success rate (N=276) and using post-procedure abdominal x-ray as opposed to fluoroscopy to assess for stent position [6]. This method allows for the patient to have definitive sepsis control in an environment with limited resources.

There are difficulties encountered when inserting a retrograde ureteric stent using local anesthesia, and although, similar to those when general anesthesia is used for the procedure, such as buckling of the stent, technical difficulties associated with a trabeculated bladder and urethral strictures and poor vision, patient tolerance of the procedure is a major limiting factor when using local anesthesia and can contribute to procedure failure. Analgesia in the form of per rectal diclofenac has been used to help with pain control [8], and Hussein et al. have suggested the use of the patient watching the procedure combined with a detailed explanation of steps to help reduce anxiety and pain and help lead to procedure success; however, more work needs to be done in the area for definitive conclusions to be drawn from this method [17].

Another limitation to this method that is worth noting is that difficult cases such as obstructed stones will increase the risk of procedure failure and thus require the patient to require further intervention in the form of ureteric stent insertion under general anesthesia or nephrostomy insertion, both of which will counter the cost and time saved using the bedside flexible cystoscopy approach.

The use of a local anesthetic and flexible cystoscopy to insert retrograde ureteric stents for early sepsis management in the form of source control has a place within healthcare, but with limited literature on this topic, further work is required to get a balanced broader view on the topic.

## Conclusions

The cases presented to demonstrate how disposable, flexible cystoscopes can be utilized in the management of septic patients by providing a means of sepsis control, at the bedside, avoiding the risks associated with both general anesthesia and potential delays getting to the emergency theater. Case selection is paramount, as is post-intervention imaging in order to increase procedure success while providing a safe alternative to the insertion of a retrograde ureteric stent in the theater.
